# “Immune Gate” of Psychopathology—The Role of Gut Derived Immune Activation in Major Psychiatric Disorders

**DOI:** 10.3389/fpsyt.2018.00205

**Published:** 2018-05-29

**Authors:** Leszek Rudzki, Agata Szulc

**Affiliations:** ^1^Department of Psychiatry, Medical University of Bialystok Bialystok, Poland; ^2^Three Towns Resource Centre, Saltcoats, United Kingdom; ^3^Department of Psychiatry, Medical University of Warsaw Warsaw, Poland

**Keywords:** microbiota-gut-brain axis, intestinal permeability, autoimmunity, psychiatric disorders, food antigens, gluten, exorphins, immunoglobulins

## Abstract

Interaction between the gastrointestinal tract (GI) and brain functions has recently become a topic of growing interest in psychiatric research. These multidirectional interactions take place in the so-called gut-brain axis or more precisely, the microbiota-gut-brain axis. The GI tract is the largest immune organ in the human body and is also the largest surface of contact with the external environment. Its functions and permeability are highly influenced by psychological stress, which are often a precipitating factor in the first episode, reoccurrence and/or deterioration of symptoms of psychiatric disorders. In recent literature there is growing evidence that increased intestinal permeability with subsequent immune activation has a major role in the pathophysiology of various psychiatric disorders. Numerous parameters measured in this context seem to be aftermaths of those mechanisms, yet at the same time they may be contributing factors for immune mediated psychopathology. For example, immune activation related to gut-derived bacterial lipopolysaccharides (LPS) or various food antigens and exorphins were reported in major depression, schizophrenia, bipolar disorder, alcoholism and autism. In this review the authors will summarize the evidence and roles of such parameters and their assessment in major psychiatric disorders.

In the last two decades, significant progress has been made in our understanding of the role of the immune system and inflammatory processes in the pathogenesis of psychiatric disorders. A recent discovery, published in NATURE (1), that the central nervous system (CNS) has its own lymphatic system is a spectacular yet thought-provoking realization; that in the vast oceans of exponentially growing amounts of scientific data, there are still major “unknowns,” which could redefine “the bigger picture.” Thanks to the synthesis of philosophy that “you cannot see the forest while looking at the leaf” along with recent fascinating discoveries of microbiotic and psychoneuroimmune complexities of the microbiota-gut-brain axis, we are now able to take a few steps back to have another, broader look at the role of the GI tract in various inflammatory, autoimmune and numerous psychiatric disorders.

The role of the GI tract in the pathogenesis of psychiatric disorders came into the scientific debate at the beginning of twentieth century (2). Buscaino reported various inflammatory changes in the GI tract in the post mortem examination of 82 patients suffering from schizophrenia. Fifty percent of those patients had manifestations of gastritis, 88% enteritis and 92% colitis (2, 3). Asperger also noted connections between celiac disease and psychotic disorders in his work (4). Baruk in his extensive work on schizophrenia pointed out the significant role of the GI tract, intestinal toxins and infection in the context of schizophrenia and catatonia (5–8). In 1979 Dohan suggested a fascinating hypothesis that “*Basic biological defect in schizophrenia is genetic impairment (e.g., via defective enzymes or, receptors) of the gut and other barrier systems which eases the passage of food-derived neuroactive polypeptides from gut lumen to brain cells”* (9). In this hypothesis he suggested that impairment of both intestinal and blood-brain-barrier (BBB) could contribute to the pathogenesis of schizophrenia.

Nowadays extensive data has revealed the indisputable role of immunity and inflammation in psychiatric disorders (10–22). The GI tract with its gut-associated lymphoid tissue (GALT) is the largest immune organ of the human organism and it produces 70–80% of immune cells. Consequently, its role in psychopathology is no longer controversial and it is drawing a lot of attention in neuroscience.

## *Stress*—the key to the “immune gate” of psychopathology

**Figure 1:**
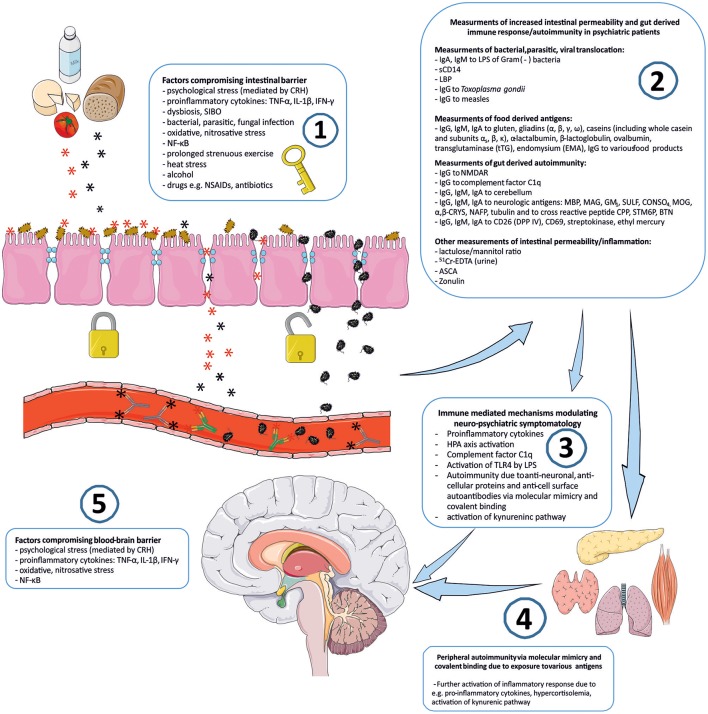
“Immune Gate” of psychopathology – mechanisms of gut derived immune activation leading to psychiatric manifestations. Image generated using Servier Medical Art. **1**: Various detrimental factors compromising intestinal barrier lead to increased intestinal permeability. **2**: Increased intestinal permeability is as a source of food derived and microbial, bacterial, parasitic antigens, and subsequent activation of inflammatory response with production of immunoglobulins against those antigens. Other markers of increased intestinal permeability. **3**: Detrimental for the brain immune consequences of gut derived antigens modulate neuro-psychiatric symptomatology. **4**: Peripheral autoimmunity (via molecular mimicry, covalent binding) due to various gut derived antigens and further activation of inflammatory response detrimental to CNS via e.g., pro-inflammatory cytokines, activation, and changes in kynurenic pathway metabolism. **5**: Factors compromising blood-brain-barrier contribute to the periphery-derived activation of inflammatory response and its psychiatric and neurological manifestations. CRH, corticoliberin; SIBO, small intestine bacterial overgrowth; NF-κB, nuclear factor kappa B; NSAIDs, non-steroidal anti-inflammatory drugs; IgA, IgE, IgG, IgM, immunoglobulin A, E, G, M; LPS, bacterial lipopolysaccharide; LBP, lipopolysaccharide binding protein; sCD14, soluble CD14; NMDAR, N-methyl-D-aspartate glutamate receptor; MBP, myelin basic protein; MAG, myelin-associated glycoprotein; GM1, ganglioside; SULF, sufatide; CONSO4, chondroitin sulfate; MOG, myelin oligodendrocyte glycoprotein; α,β-CRYS, α,β-crystallin; NAFP, neurofilament proteins; CPP, Chlamydia pneumoniae; STM6P, streptococcal M protein; BTN, milk butyrophilin; DPP IV, dipeptidyl peptidase IV (synonym of CD26); _51_Cr-EDTA, chromium ethylenediaminetetraacetic acid; ASCA, IgG to *Saccharomyces cerevisiae*; TLR4, Toll-like receptor 4.

Connotation of the word *Stress* usually relates to its psychological perspective. It is mostly perceived as the feeling of fear, threat, anger, frustration, hatred, insecurity, abandonment, and unpredictability. Stress reaction may also take the form of the fight-flight-freeze response. However, stress is non-specific and for the human organism it has a much broader meaning. Inflammation, viral, bacterial or parasitic infections, injury, exposure to various toxins, radiation, oxidative and nitrosative stress, and excessive physical training are also recognized as stress by the human organism. The body's reaction to various stressors is relatively uniform, whether it is facing psychological or physical stressors. On one hand, stress may activate the immune system and inflammatory response, e.g., via an elevated level of pro-inflammatory cytokines, and the trafficking of immune cells between blood and tissues. This activation is preparing the organism to “face and fight” potential threats. On the other hand, stress response leads to the activation of the hypothalamic–pituitary–adrenal axis (HPA) and to the increased secretion of anti-inflammatory adrenal hormone, cortisol. This “safety switch” is supposed to prevent an excessive activation of potentially destructive inflammatory response (23–25). Interestingly, all of the stressors mentioned above can directly or indirectly lead to increased intestinal permeability and its various immune and psychopathological consequences. The GI tract forms the largest surface, about 300 m^2^, of interaction between the internal and external environment of the human body (26). The intestinal barrier constitutes of one layer epithelium composed of enterocytes interconnected by protein junctional complexes—tight junctions (zonulae occludentes). Permeation of molecules from the intestinal lumen is both transcellular and paracellular, and the opening of tight junctions regulate the latter (27). Moreover, the mucosal layer and intestinal microbiota are also crucial elements of this barrier and they are determining its permeability (28, 29). Psychological stress mediated by corticoliberin (CRH) (26, 30–33), proinflammatory cytokines e.g., IL-1β (34), TNF-α (35, 36), INF-γ (37), dysbiosis (38, 39), small intestine bacterial overgrowth (SIBO) (40), bacterial, parasitic or fungal infections (41, 42), oxidative and nitrosative stress (32, 43), the nuclear factor NF-κB (44), prolonged strenuous exercise (45, 46), heat stress (47), alcohol (38, 48–50), food additives (51), certain drugs e.g., non-steroidal anti-inflammatory drugs (NSAIDs) (52, 53) or antibiotics (54–57) can cause the loss of intestinal barrier integrity leading to its excessive permeability (Figure 1). This may lead to subsequent immune activation with further consequences in the CNS (39). It is worth mentioning that the structure and mechanics of the BBB are in many ways similar to the GI barrier (58), and BBB permeability may also be compromised by analogous factors to those compromising the GI barrier e.g., psychological stress (59), pro-inflammatory cytokines (60–66), oxidative and nitrosative stress (63, 67, 68) (Figure 1).

There are various mechanisms for how increased intestinal permeability and gut-derived antigens can have immune-mediated consequences for the brain and behavior. Measurements of these mechanisms could serve as potential biomarkers for the involvement of the gut-brain axis in the psychopathology of patients and could also have significant value in broader therapeutic approach.

## Bacterial translocation

Bacterial lipopolysaccharides—LPS (a glycolipid complexes found in the outer membrane of Gram negative bacteria) have a profound influence on immunity and brain function, and upstream activation of Toll-like receptor 4 (TLR4) by LPS has a crucial role in these interactions (69, 70). Therefore, LPS challenge represents a laboratory model of inducing transient, low-grade inflammation with subsequent behavioral changes in animals and sickness behaviors in human subjects (71). Usually this challenge is performed through intravenous or intramuscular injection of LPS. However, some of the major consequences of the various stressors discussed above, including psychological stress, are dysfunction of the gut barrier, increased permeability, translocation of enteric bacteria from the intestinal lumen, and subsequent activation of the inflammatory response by LPS (29) (Figure 1). It was demonstrated that chronic psychological stress increased intestinal permeability of *E. coli* via follicle associated epithelium, by more than 30-fold and permeability to the antigenic protein horseradish peroxide (HRP), almost 4-fold. Moreover, serum corticosterone was significantly increased after 10 days of chronic stress and this was accompanied by a 3-fold increase in the number of mucosal mast cells. In this study acute stress also increased permeability to HRP (72). Bacterial LPS are able to induce enzymes of the kynurenic pathway, e.g., indoleamine 2,3 dioxygenase (IDO), the first step enzyme converting tryptophan toward the kynurenine pathway. It was demonstrated in animal studies that peripheral LPS challenge resulted in increased expression of brain pro-inflammatory cytokines and neuroinflammatory glial cellular markers. This was also accompanied with increased activity of brain kynurenic pathway enzymes with subsequent activation of neurotoxic branch of this pathway toward synthesis of detrimental kynurenines (73). In healthy humans administration of LPS resulted in increased body temperature, malaise, increased levels of cortisol and pro-inflammatory cytokines e.g., TNF-α, soluble TNF receptors, IL-6, IL-1, and IL-1 receptor antagonist. Furthermore, this low-grade immune response was accompanied by increased anxiety, depressed mood and a decrease in verbal and non-verbal memory functions (74, 75). Also, administering bacterial LPS had a dose dependent negative impact on cognitive functions (76). Moreover, bacterial translocation and LPS are known to induce monocyte activation and their trafficking into the CNS. This is considered to be a crucial mechanism of action in the pathogenesis of human immunodeficiency virus (HIV)-associated dementia (77).

Measurements of antibodies against LPS of Gram negative enterobacteria can be used as surrogate marker for the assessment of intestinal permeability. Due to increased intestinal permeability we can expect increased bacterial translocation and increased blood concertation of immunoglobulins against various enterobacteria. This approach was initially used by Maes et al. in assessing patients with chronic fatigue syndrome (CFS) (78) (Table 1). Patients suffering from this disorder had increased prevalence and median values of serum IgA against LPS of enterobacteria compared to those of healthy controls and patients with partial CFS. Additionally, IgA levels were significantly correlated to the severity of CFS, such as irritable bowel, muscular tension, fatigue, the inability to concentrate and failing memory. These results suggest that increased intestinal permeability to enterobacteria are involved in the etiology of CFS. In another study patients with CFS had increased levels of LPS, along with elevated levels of surrogate markers of bacterial translocation namely, soluble CD14 (sCD14) and lipopolysaccharide binding protein (LBP) (79). In the same study, profiling of gut microbial diversity by sequencing 16s ribosomal ribonucleic acid (rRNA) genes from stool, revealed reduction in diversity and abundance of bacteria belonging to Firmicutes phylum, and reduction of anti-inflammatory with the concurrent increase of pro-inflammatory bacterial species in CFS patients. Results of this study indicate that besides increased intestinal microbial translocation, dysbiosis of gut microflora may also play a role in inflammatory symptoms of CFS.

**Table 1:** Major research on the gut derived immunity in psychiatric disorders.

**Measured parameter**	**References**	**Number of participants**	**Psychiatric diagnosis**	**Major findings**	**Major conclusions**
Surrogate marker of intestinal permeability: serum concentrations of IgA and IgM against LPS of Gram (-) enterobacteria: *Hafnia alvei, Pseudomonas aeruginosa, Morganella morganii, Proteus mirabilis, Pseudomonas putida, Citrobacter koseri, Klebsiella pneumonia*.	(78)	CFS *n* = 29 Controls *n* = 11	CFS	↑ Prevalence and median values for serum IgA against the LPS of enterobacteria in CFS compared to controls and patients with partial CFS. Serum IgA levels correlated to the severity of CFS such as irritable bowel, muscular tension, fatigue, concentration difficulties, failing memory.	Enterobacteria are involved in the aetiology of CFS and increased gut permeability caused an immune response to the LPS.
LPS Surrogate markers of bacterial translocation: soluble CD14 (sCD14) and lipopolysaccharide binding protein (LBP). Profiling gut microbial diversity by sequencing 16s ribosomal ribonucleic acid (rRNA) genes from stool.	(79)	CFS *n* = 48 Controls *n* = 39	CFS	↑ LPS, LBP, sCD14 in CFS patients. LBP levels correlated with LPS and sCD14 LPS correlated with sCD14 Bacterial diversity was reduced in CFS patients, in particular, reduction in diversity and abundance of bacteria belonging to Firmicutes phylum In CFS reduced anti-inflammatory and increased pro-inflammatory bacterial species.	↑ intestinal microbial translocation and dysbiosis of gut microflora in may play a role in inflammatory symptoms of CFS.
Serum concentration of IgM and IgA against LPS of enterobacteria: *H. alvei, P. aeruginosa, M. morganii, P. putida, C. koseri, K. pneumoniae*.	(80)	MDD *n* = 28 Controls *n* = 23	MDD	↑ IgM levels against LPS of *P. aeruginosa and P. putida* in MDD. ↑ The peak IgM, IgA and the total sum of all six IgM and IgA values in MDD. The symptom profiles of ↑ IgM and IgA were: fatigue, autonomic and gastro-intestinal symptoms, and subjective feeling of infection.	↑ translocation of Gram (–) bacteria (“leaky gut”) may play a role in inflammatory pathophysiology of MDD.
Serum concentration of IgM and IgA against the LPS of enterobacteria: *H. alvei, P. aeruginosa, M. morganii, P. putida, C. koseri, K. pneumoniae*.	(81)	MDD *n* = 112 Controls *n* = 28	MDD	↑ IgM against *H. alvei, P. aeruginosa, M. morganii and P. putida* in MDD. ↑ IgA against *H. alvei, P. aeruginosa, M. morganii, K. pneumonia* in MDD. ↑ Peak IgM and IgA responses in MDD. ↑ Peak IgM responses in chronic MDD versus non-chronic MDD. Significant differences in IgM responses between patients with chronic MDD (duration > 2 years) and controls.	Increased translocation of Gram (–) bacteria may play a role in inflammatory pathophysiology of (chronic) MDD.
sCD14 LBP	(82)	SZ *n* = 141; BD *n* = 75; Controls *n* = 78; Antipsychotic naïve 1st episode of SZ *n* = 78; Medicated 1st episode of SZ *n* = 38	SZ, BD	sCD14 seropositivity conferred a 3.1-fold ↑ odds of association with schizophrenia. LBP correlated with BMI in schizophrenia. In bipolar disorder sCD14 levels correlated with anti-transglutaminase IgG.	↑ intestinal permeability to Gram (–) bacteria could contribute to the results. Non-LPS related monocyte activation, autoimmunity, and metabolic profiles could also contribute to the results.
Measurement of intestinal permeability ^51^Cr-EDTA (urine) Plasma LPS concentration Plasma TNFα, IL-6, IL-10, hsCRP (measurements performed at the beginning T1 of the 3 week detoxification programme and after the treatment – T2)	(50)	Patients *n* = 26 Controls *n* = 16	Alcohol dependence	↑^51^Cr-EDTA in patients versus controls at T1 and no difference at T2. ↓Intestinal permeability in patients during the treatment. ↑Plasma LPS in patients versus controls at T1, significantly ↓ during withdrawal and no difference from controls at T2. A low-grade inflammation observed at T1 and partially ↓ during withdrawal. At T1 pro-inflammatory cytokines significantly correlated with craving. At T2 anti-inflammatory IL-10 negatively correlated with depression, anxiety and craving.	“Leaky gut” and the gut–brain axis may play a role in the pathogenesis of alcohol-dependence.
IgA and IgG to gluten, gliadin, casein, α-lactalbumin, β-lactoglobulin, ovalbumin	(83)	SZ *n* = 48 (medicated) Historical control group SZ *n* = 13 (drug free) Controls *n* = 13	SZ	↑ in SZ of IgA to gliadin, β-lactoglobulin, casein compared to controls. No significant difference for the IgG data.	More patients with schizophrenia than controls showed IgA antibody levels above the upper normal limit to gliadin, beta-lactoglobulin, and casein.
IgA and IgG to gliadin IgA to transglutaminase (tTG) IgA to EMA	(84)	SZ *n* = 1,401 Controls *n* = 900	SZ	23.1% of patients had moderate to high levels IgA to gliadin compared with 3.1% in control group. 5.4% of patients had moderate to high levels of IgA to tTG compared to 0.80% in control group. Only 0.35% (*n* = 5) patients were positive for IgA to EMA.	Patients with SZ have ↑ levels of antibodies related to CD and gluten sensitivity. There is a specific immune response to gluten in SZ.
CSF levels of opioid receptor-active, endorphin fraction (Fraction I) CSF levels of monoamine metabolites	(85)	SZ *n* = 45 Controls *n* = 18	SZ	↑levels of Fraction I in SZ compared to controls ↑levels of Fraction I in SZ were associated with low levels of the dopamine metabolite homovanillic acid in drug-free SZ patients	There is ↑ opioid activity and concomitant dysfunction of brain endorphin and dopamine activity in SZ patients.
CSF levels of opioid receptor-active components (fraction II activity)	(86)	PP *n* = 11 Lactating controls *n* = 11 Non lactating controls *n* = 16	Postpartum psychosis	Very high levels of fraction II activity (bovine beta-casomorphin) were observed in four PP patients	Certain cases of PP are associated with the occurrence in plasma and CSF of unique opioid peptides related to bovine beta-casomorphin.
IgG and IgA to gliadin and tTG IgG to delaminated gliadin HLA DQ2 and HLA DQ8 alleles assessment	(87)	Recent onset psychosis *n* = 129 Multi episode SZ *n* = 191 Controls *n* = 151	Recent onset psychosis, SZ	↑ IgG and IgA to gliadin in recent-onset psychosis. ↑ IgG and IgA to gliadin in patients with multi-episode schizophrenia but lower than in recent onset. IgG to deamidated gliadin and IgA to tissue to tTG not elevated in either group. Fewer than 1% individuals in each of the groups had levels of these antibodies predictive to celiac disease No differences in the distribution of the HLA DQ2 and HLADQ8 among groups.	There might be a common immunologic feature similar to celiac diseases in patients with schizophrenia which have increased antibodies levels to gliadin.
IgG to whole casein and to the α_s_, β, κ casein subunits	(88)	Recent onset psychosis *n* = 95 Long-term SZ *n* = 103 Controls *n* = 65	Recent onset psychosis, SZ	↑ IgG to whole casein proteins, α_s_, β and κ subunits in recent onset of psychosis. In this group odds ratio particularly significant for psychotic disorders with depressive symptoms. ↑ IgG to whole casein and α_s_ subunit in long-term schizophrenia. PANNS scores for negative symptoms significantly correlated with casein antibody levels for the α_s_ and κ subunits.	Current results provide a rationale for performing clinical trials of dietary interventions in psychiatric patients.
IgG to *Saccharomyces cerevisiae* (ASCA – marker of intestinal inflammation) IgG to bovine milk casein, wheat-derived gluten IgG to *Toxoplasma gondii*, EBV, Influenza A, Influenza B, Measles, Rubella	(89)	Non-recent onset *n* = 193 Recent onset *n* = 67 1st episode *n* = 103 (including 40 antipsychotic-naïve) Controls *n* = 207	SZ	↑ASCA IgG and correlated with food antigen antibodies in recent onset and non-recent onset schizophrenia compared to controls. ↑ASCA IgG in unmediated patients with first episode of schizophrenia compared to patients receiving antipsychotic treatment. In the recent onset group significant correlation of IgG to casein with IgG to *T. gondii* and significant correlation of IgG to gluten with IgG to *T. gondii*.	Inflammation and changes in GI permeability may contribute to etiopathogenesis and/or symptomatology of schizophrenia. GI inflammation may occur in the absence of antipsychotics and may be modified by them.
ASCA IgG to casein and gluten IgG to *T. gondii* sCD14 **Mouse model:** IgG to casein and gluten IgG to *T. gondii* Complement system Anti-NMDA receptor antibodies	(42)	SZ *n* = 263 Controls *n* = 207	SZ	↑ASCA IgG and correlated with - IgG to casein and gluten in SZ. ↑ CD14 in SZ. IgG to *T.gondii* correlated with IgG to casein and gluten in SZ. **Mouse model:** *T.gondii* infection may result with ↑ of IgG to casein and gluten, ↑ of complement factors and ↑ of autoantibodies to the brain NMDA receptors.	Intestinal inflammation and ↑ intestinal permeability are relevant in pathology of schizophrenia. Infection with *T. gondii* may play a role in pathology of schizophrenia and autoimmunity against NMDA receptors.
IgG to human complement factor C1q	(90)	Non-recent onset of SZ *n* = 61 Recent onset of SZ *n* = 38 Controls *n* = 63	SZ	C1q IgG levels were highest in recent-onset SZ and moderately elevated in non-recent onset of SZ. ↑ Casein and/or gluten-IgG binding to C1q in the non-recent onset. Significant associations of immune complex seropositivity with the non-recent onset group. C1q IgG antibody levels associated with casein IgG, gliadin IgG and ASCA IgG.	Complement activation may be a useful marker in schizophrenia during early stages of the disease.
IgG to gluten and casein in serum and cerebrospinal fluid (CSF)	(91)	1st episode SZ *n* = 105 including *n* = 75 antipsychotic-naïve Controls *n* = 61	SZ	Striking correlations of IgG response to dietary proteins between serum and CSF in patients but not in controls. ↑ parameters of the blood-CSF permeability, the CSF-to serum albumin ratio in SZ. Lack of evidence for the intrathecal production of the food-related IgG within the CNS CSF IgG index and specific Antibody Index) in SZ.	Patients with SZ may have dysfunction/increased permeability of blood-brain-barrier or/and blood-CSF barrier. Those could be the ways of entering casein and gluten IgG to CNS with subsequent role in brain pathology.
IgG and IgA to gliadin IgG and IgA to tTG IgG to deamidated gliadin	(92)	BD *n* = 102 Controls *n* = 173	BD	↑ IgG to gliadin and to deamidated gliadin in BD. IgA antigliadin antibodies and antibodies to tTG did not differ between groups.	Patients with BD have ↑ levels of antibodies to gliadin. There is no elevation of other antibodies typical for CD. Possible another pattern of antibody response to gluten in BD.
Longitudinal assessment with follow-up 6 months later of: Serum IgG and IgA to gliadin IgG and IgA to tTG IgG to deamidated gliadin	(93)	Mania *n* = 60 Controls *n* = 143	Mania in course of BD I, II, schizoaffective disorder.	↑ IgG to gliadin but not other markers of celiac disease in mania at baseline. At the 6 months follow up no difference of above parameters from controls. ↑ IgG to gliadin at follow-up significantly associated with re-hospitalization in the 6 months follow-up period.	The monitoring and assessment of gluten sensitivity may have be significant in the management of patients with acute mania.
IgG to ASCA IgG to bovine milk casein IgG to wheat gluten EBV IgG, Influenza A IgG, Influenza B IgG, Measles IgG, *Toxoplasma gondii* IgG	(94)	BD without a recent onset of psychosis *n* = 226 BD with recent onset of psychosis *n* = 38 Controls *n* = 207	BD	↑ ASCA IgG in both groups of BD. ↑ IgG to casein and gluten in both BD groups. ASCA IgG correlated with IgG to casein and gluten in both BD groups. ASCA IgG correlated with measles and *T.gonii* in BD with recent onset of psychosis. In BD without a recent onset of psychosis ASCA IgG correlated with IgG to casein and gluten in manic, depressed or mixed episodes subgroups. In BD with recent onset of psychosis ASCA IgG correlated with IgG to casein and gluten in manic subgroup. No influence of medication on ASCA IgG.	Results are strong preliminary evidence for a role of GI tract in the inflammatory pathology of BD. Treatment strategies involving diet modifications, anti-inflammatory agents and microbiota modulations should be further investigated.
IgG to 44 different food products Cortisol, IL-1b. IL-6, TNFα	(95)	Patients *n* = 34 Controls *n* = 29	MDD	Significant positive correlations of IgG to 11.36% food products and length of depressive episode (months). No significant differences in mean IgG concentrations against 44 food antigens between patients and controls. ↓ IgG concentration to dairy in depressed patients compared to controls in subgroups with high exposure (consumption) of diary.	No differences in mean IgG to food antigens, however positive correlations between the length of depressive episode with IgG concentrations to food antigens suggest that further research in recurrent, chronic depression would be valuable.
IgA to ASCA IgG to gliadin IgA to LPS CRP	(96)	Patients *n* = 210 Controls *n* = 72	SZ, BD, MDD. 10% patients “s” attempt in last month. 45% patients “s” attempt in their lifetime.	↑ IgA to ASCA, IgG to gliadin and IgA to LPS in recent suicide attempters (last month) compared to controls. Those markers were no elevated in patients with past, but not recent, suicidal history.	GI inflammation may be associated with recent suicidal attempt and should be further explored as a predictive marker of such attempts.
Intestinal permeability measurement (lactulose/mannitol ratio – LA/MA) IgA to tTG, EMA Total mucosal IgA HLA-DQ2/-DQ8 haplotypes Total IgA, IgG, IgE IgA and IgG to α-gliadin IgA and IgG to deamidated gliadin IgG to gliadins α, β, γ, ω IgG to β-lactoglobulin, α-lactalbumin, casein IgE to milk, casein, gluten, lactoglobulin, α-lactalbumin	(97)	Patients *n* = 162 Controls *n* = 44	ASD	↑ intestinal permeability (LA/MA) 25.6% of ASD patients compared to 2.3% of controls ↑ IgG to AGA and deamidated gliadin in ASD. ↑ IgG to casein in ASD.	Immune system is triggered by gluten and casein in ASD patients and impaired intestinal barrier could contribute to that.
IgG, IgA, IgM to casein, lactalbumin, β-lactoglobulin, ovalbumin Assessment of behavioral symptoms after 8 weeks of elimination diet	(98)	Patients *n* = 36 Controls *n* = 20	ASD	Improvement of behavioral symptoms of patients after 8 weeks of elimination diet. ↑ IgA to casein, lactalbumin, β-lactoglobulin in ASD. ↑ IgG and IgM to casein in ASD. ↑ of positive skin prick test in ASD. ↑ IgE levels and skin tests and specific IgE more frequent for casein, lactalbumin, β-lactoglobulin, egg white, rice, and soy.	Results suggest relationship between food allergy and infantile ASD.
Zonulin	(99)	Patients *n* = 32 Controls *n* = 33	ASD	↑ serum zonulin in patients compared to controls Positive correlation between zonulin levels and Childhood Autism Rating Scale.	Zonulin, regulator of gut permeability, plays a role in development of ASD.
*Post-mortem* measurement of gene and protein expression of brain (cortex, cerebellum) proteins and key molecules associated with BBB and tight junctions, neurovascular unit integrity and neuroinflammation. Gene and protein expression of intestinal tight junctions in duodenal biopsies.	(100)	Brain *post –mortem* samples: ASD *n* = 8 SZ *n* = 10 Controls *n* = 15 Duodenal biopsies: ASD *n* = 12 Controls *n* = 9	ASD, SZ	↑Claudin−5 and−12 in ASD cortex and cerebellum. ↑Claudin−5, tricellulin, MMP-9 in ASD cortex. ↓IL-8, tPA, IBA-1 in SZ cortex. ↑IL-1b in SZ cerebellum. ↓Claudin−12 in ASD and SZ cortexes. ↓ expression of components of intestinal tight junctions (claudin-1, occludin and tricellulin) in 75% of ASD patients ↑ intestinal pore-forming claudins (claudin-2,−10,−15) in 66% of ASD patients compared to controls.	In brain of patients with ASD there is an altered expression of genes related to blood-brain-barrier integrity coupled with elevated neuroinflammation and possibly impaired gut barrier integrity.
Simultaneous presence of IgG, IgM, IgA antibodies to gliadin and cerebellum Examining cross-reaction between dietary proteins and cerebellar antigens	(101)	Patients *n* = 50 Controls *n* = 50	ASD	Concomitant ↑ IgG, IgM, IgA to gliadin and cerebellum in more than 80% of patients. Demonstrated cross-reactivity between gliadin and cerebellar peptides.	Subgroup of patients with ASD produce antibodies against cerebellar Purkinje cells and gliadin peptides which may be responsible for some of the neurological symptoms in ASD.
IgG, IgM, IgA antibodies to neurologic antigens: myelin basic protein (MBP), myelin-associated glycoprotein (MAG), ganglioside (GM_1_), sufatide (SULF), chondroitin sulfate (CONSO_4_), myelin oligodendrocyte glycoprotein (MOG), α,β-crystallin (α,β-CRYS), neurofilament proteins (NAFP), tubulin Cross reactive peptides: *Chlamydia pneumoniae* (CPP), streptococcal M protein (STM6P), milk butyrophilin (BTN).	(102)	Patients *n* = 40 Controls *n* = 40	ASD	ASD patients showed the highest levels of IgG, IgM, IgA against all neurologic antigens as well as the three cross-reactive peptides.	Neurologic antibodies may have been synthesized due to alterations in BBB. These results suggest mechanisms by which bacterial infections and milk antigens modulate autoimmune response in ASD.
IgG, IgM, IgA antibodies to CD26 (DPP IV), CD69, streptokinase (SK), gliadin, casein, ethyl mercury. Assessment of binding of SK, gliadin, casein and ethyl mercury with CD26 and CD69.	(103)	Patients *n* = 50 Controls *n* = 50	ASD	Significant percentage of ASD children developed anti-SK, anti-gliadin and casein, anti-ethyl mercury antibodies concomitantly with anti-CD26, anti CD-69 autoantibodies. Adding SK, gliadin, casein, ethyl mercury to CD26 or CD69 resulted in 28–86% inhibition of CD26 or CD69 binding to anti-CD26 and anti CD-69 antibodies.	First demonstration that dietary peptides, bacterial toxins and xenobiotics bind to lymphocyte receptors and/or tissue enzymes, resulting in autoimmune reaction in ASD.

*ASCA, IgG to Saccharomyces cerevisiae; ASD, autism spectrum disorder; BP, bipolar disorder; CD, celiac disease; ^51^Cr-EDTA, chromium ethylenediaminetetraacetic acid; CRP, C reactive protein; CFS, chronic fatigue syndrome; DPP IV, dipeptidyl peptidase IV (synonym of CD26); EBV, Epstein-Barr Virus; EMA, endomysium; HLA DQ2, DQ8, human leukocyte antigens DQ2, DQ8; IgA, IgE, IgG, IgM, immunoglobulin A, E, G, M; LA/MA, lactulose/mannitol ratio; LBP, lipopolysaccharide binding protein; LPS, bacterial lipopolysaccharide; MDD, major depression; PANNS, The positive and negative syndrome scale; “s” attempt, suicidal attempt; sCD14, soluble CD14; SZ, schizophrenia; PP, postpartum psychosis; T. gondii, Toxoplasma gondii*.

Furthermore, the role of increased intestinal permeability to enteric bacteria, also known as “leaky gut,” was demonstrated in two consecutive studies and it was suggested to be a potential pathophysiological mechanism of major depression (80, 81). In these studies (Table 1) serum concentrations of IgM and IgA were measured against LPS of six different enterobacteria: *Hafnia alvei, Pseudomonas aeruginosa, Morganella morganii, Pseudomonas putida, Citrobacter koseri*, and *Klebsiella pneumonia*. Levels of immunoglobulins were significantly higher in depressed patients compared to the control group. Interestingly there were also significantly higher IgM responses in patients suffering from chronic depression (duration > 2 years) when compared to patients with non-chronic depression and controls. This suggests that patients with chronic depression may also have increased intestinal permeability to a larger extent than non-chronic patients. This conclusion seems to be particularly valuable in further understanding of the mechanisms of treatment-resistant and chronic depression.

Excess alcohol consumption is a known factor for compromising the gut barrier, leading to increased intestinal permeability to macromolecules and bacterial endotoxins (38, 48–50). Furthermore, translocation of gut-derived bacterial toxins and related inflammatory response are believed to play a significant role in the development of progressive alcoholic liver injury (104–106). In alcohol-dependent patients, a 3 week alcohol detoxification programme resulted in significant improvement in intestinal barrier function, assessed using urine ^51^Cr-EDTA and plasma LPS levels (50). Additionally, parameters of systemic inflammations (TNF-α, IL-6, IL-10, hsCRP) partially decreased during withdrawal and inflammatory parameters correlated with depressive symptoms and alcohol-craving (Table 1). These results suggest that the gut-brain axis could also have significance in the pathogenesis of alcohol dependence and in affective symptomatology observed in this disorder.

## Food derived antigens in psychiatric disorders

Another group of parameters believed to be contributing factors in psychopathology are related to food derived antigens and exorphins (Figure 1). The current literature covering this topic is mostly focused on the involvement of glutens, caseins, and exorphins in disorders such as psychoses, schizophrenia, bipolar disorder, and autistic spectrum disorders (ASD) (Table 1). Glutens are storage proteins of various grass-related grains. They consist of numerous protein components and can be divided into two main fractions: the soluble in aqueous alcohols gliadins, and insoluble glutenins (107). Bovine milk is another source of food derived antigens and it consists of two major group of proteins: caseins and whey proteins. Caseins account for 80% of bovine milk proteins and they are divided into a further four major groups: α_s1_, α_s2_, β, and κ caseins (108). Some milk and gluten proteins can be a source of exorphins, which are peptides with morphine-like activity due to their ability to bind to opioid μ-receptors e.g., in the CNS or gastrointestinal tract (109). Moreover, exorphins can stimulate T-cells and induce peptide-specific T cell responses that may result in further activation of inflammation, including elevated concentrations of pro-inflammatory cytokines and autoimmunity (103). Furthermore, in animal study, consumption of β-casein derived peptides, β-casomorphins, resulted with inflammatory immune response in gut (110). Thus, food-derived compounds may influence immunity and brain function due to their antigenic, pro-inflammatory qualities and/or their abilities to behave as ligands of various opioid receptors. Previously high levels of β-casomorphin-like opioid peptides were observed in CSF and serum of patients with postpartum psychosis (86). Also, patients with schizophrenia had increased opioid activity in CSF (85) and exorphins were found in the urine of untreated patients with schizoaffective disorder (111).

Numerous studies report that patients experiencing psychiatric and neurologic symptoms have abnormal reactions to food-derived antigens. For instance, celiac disease (CD)—an autoimmune disorder and widely recognized manifestation of gluten sensitivity, has various psychiatric, and neurologic manifestations and is considered to be a gut-brain axis “flagship” condition (112, 113). Various epidemiological studies have shown substantial association of schizophrenia with CD (114–116). However, there are also various examples of abnormal responses to food antigens which go beyond the presence of antibodies to deamidated epitopes of gliadin and tissue transglutaminase (tTG) which are characteristic immune responses observed in CD (117). It was previously demonstrated that patients with schizophrenia had increased IgA to gliadin, β-lactoglobulin and casein (83). A large study of 1401 schizophrenia patients from the CATIE study (clinical Antipsychotic Trials of Intervention Effectiveness), and 900 controls, revealed that 23.1% of patients had moderate-to-high levels of IgA to gliadin (IgA-AGA) compared with 3.1% in the control group (84). Moderate-to-high levels of antibodies to tTG were also observed in 5.4% of patients with schizophrenia compared with the 0.80% in the control group. Only 0.35% (*n* = 5) patients were positive for IgA to endomysium (EMA). Results of this study revealed that patients with schizophrenia have higher levels of antibodies related to CD and gluten sensitivity and that there is also a specific immune response to gluten in this population. In another study, patients with the recent-onset of psychosis and patients with multi-episode schizophrenia had increased levels of IgG and IgA antibodies to gliadin compared with controls (87). However, these patients did not have increased IgG to deamidated gliadin or IgA antibodies to tTG, and < 1% of patients had levels of antibodies symptomatic for CD. These results point to the existence of different immune mechanisms in schizophrenia compared to those observed in CD. Moreover, increased IgA antibody levels to gliadin, β-lactoglobulin, and casein were observed in schizophrenia (83). Elevated IgG to whole casein and α_s_, β, κ casein subunits was demonstrated in patients with recent-onset of psychosis. In contrast, in the group suffering from long-term schizophrenia there was an increase in IgG to whole casein and α_s_ subunit (88). Interestingly, in this study Positive and Negative Syndrome Scale (PANNS) scores for negative symptoms significantly correlated with casein antibody concentration to subunits α and β. In recent years, a novel syndrome of gluten intolerance, known as non-celiac gluten sensitivity (NCGS) or gluten sensitivity (GS), has gained recognition (118). Patients with NCGS do not develop typical antibodies of CD, however they experience various physical and behavioral symptoms after gluten consumption. The most common symptoms are IBS-like symptoms, chronic fatigue, headache, bone and joint pain, numbness of hand and feet, erythema, muscle contractions, and depression. Patients may also experience hyperactivity, disturbed attention and it is likely that NCGS may contribute to symptoms of other psychiatric disorders (119).

Anti-*Saccharomyces cervisiae* IgG antibodies (ASCA), typically increased in Crohn's disease or ulcerative colitis, is a marker of GI inflammation (94). It was demonstrated that levels of ASCA IgG were significantly elevated in patients with schizophrenia compared to the control group, and ASCA significantly correlated with antibody levels to gluten and casein in the same patients. Interestingly, in this study authors revealed significant correlations between IgG to *Toxoplasma gondii* and IgG to food antigens in recent-onset schizophrenia. They suggested that infection with this parasite could result in increased permeability of the intestinal barrier with subsequent increased absorption of food antigens (89). Infection with *T. gondii* is also a known risk factor for the development of schizophrenia. Severance et al. demonstrated a fascinating association between *T. gondii* infection with gut-derived inflammation, increased intestinal permeability, allergy to food antigens and development of anti-NMDA receptor autoantibodies (42). In this study, patients with schizophrenia had increased levels of ASCA, which correlated with antibody levels to gluten and casein. Moreover, these patients had increased levels of soluble CD14—a marker of intestinal microbial translocation previously mentioned above. Infection with *T. gondii* also correlated with antibodies to food antigens. In further investigation of these clinical observations, using a mouse model, the same authors demonstrated that *T. gondii* infection may result in the elevation of IgG to casein and gluten, activation of complement system and increased levels of autoantibodies to the brain NMDA receptors.

Another group of molecules receiving a lot of attention in psychiatry research and neuroscience recently is the complement system of the immune system. The complement system is a protein complex involved in the recognition, opsonisation and lysis of various antigens. These proteins are also involved in synapse development, neuronal pruning and neurodegeneration, and are present in the human CNS, where they are mostly produced by activated microglia (120–122). Involvement of the complement system was demonstrated in various neurodegenerative disorders such as Alzheimer's disease (123, 124), Huntington's disease (125), Parkinson's disease (126), Pick's disease (127) and amyotrophic lateral sclerosis (ALS) (128). Moreover, activation of the complement system has been demonstrated in schizophrenia (129–133) and autism (134–136), and it is believed that the complement system could contribute to symptomatology of those disorders. For instance, association of C1qB gene polymorphism with schizophrenia was demonstrated in an Armenian population and it was suggested that the“C1qB gene may be considered as a relevant candidate for susceptibility to schizophrenia.” Interestingly, Severance et al. suggested the hypothesis that food antigens could be the source of activation of the complement system, and that these antigens could bind and activate the C1q, the first component in classical activation of the complement system. These authors demonstrated increased binding of casein and/or gluten IgG to C1q in patients with non-recent onset schizophrenia compared to controls and that levels of C1q-casein/gluten-related immune complexes and C1q correlated with ASCA. The authors of this study suggested “complement activation may be a useful biomarker to diagnose schizophrenia early during the course of the disease” (90). Furthermore, an upregulation of cerebral C1q was demonstrated in response to latent *T. gondii* infection and it was hypothesized that “complement activity may aid in the clearance of this parasite from the CNS and in so doing, have consequences for the connectivity of neighboring cells and synapses” (137).

As suggested by Dohan in 1979, besides increased permeability of intestinal barrier, dysfunction of barrier systems within the CNS could be another contributing factor for heightened transit of food-derived antigens and neuroactive polypeptides from the intestinal lumen to the CNS (9, 138). In line with this hypothesis, striking correlations between serum and cerebrospinal fluid (CSF) IgG to wheat gluten and bovine milk casein were demonstrated in antipsychotic-naïve schizophrenia patients compared to healthy controls (91). In the same study, there was a lack of intrathecal, local CNS production of IgG to food antigens, which supported the hypothesis that these antigens were derived from the periphery and were required to cross to the CNS via defective BBB (91). Previously the dysfunction of BBB was also reported in psychotic and affective disorders, and autism (100, 139–141).

Bipolar affective disorder is another severe psychiatric disorder in which compromised gut barrier has been demonstrated (Table 1) (92–94, 96). Patients with this disorder had elevated serum concentrations of IgG to gliadin and deamidated gliadin in comparison to controls. There was no difference in IgA to gliadin and to tTG between patients and control group (92). In a follow-up study, patients with manic symptoms had increased baseline IgG to gliadin, which normalized after 6 months of treatment (93). In the same study, re-hospitalized patients during a 6-month follow-up period were more likely to have increased IgG to gliadin at the follow-up. Analogically to schizophrenia there is also evidence for increased GI inflammatory parameters in patients with bipolar disorder. Patients with this disorder were demonstrated to have increased levels of ASCA along with IgG to casein and gluten, and ASCA correlated with IgG to these food antigens compared to controls (94). ASCA were also correlated with IgG to *T. gondii* and measles in patients who experienced recent-onset of psychosis in the course of bipolar disorder.

In a study performed by our group, we measured IgG against 44 different food products in patients with Major Depressive Disorder (MDD). We found significant positive correlations of IgG to 11.36% of food products with the length of depressive episode (months). We did not observe significant differences in mean IgG concentrations against 44 food antigens between patients and the control group, however most of our patients experienced the first episode of MDD, which could have significantly influenced our results. The conclusion of the study was that “it could be valuable to further explore a potential role for increased intestinal permeability to food antigens with subsequent IgG responses in patients with chronic, recurrent depression, and in patients with gastrointestinal, and extra-intestinal autoimmune diseases with co-morbid depression” (118).

GI inflammation and increased intestinal permeability may play also a significant role in suicidal symptomatology. In a recent pilot study it was demonstrated that recent suicidal attempters (within the last month) in the course of major depression, bipolar disorder and schizophrenia had increased IgA to ASCA, IgG to gliadin and increased IgA to LPS compared to a healthy control group (96). Moreover, association between the number of suicide attempts and the levels of IgM antibodies to *T. gondii* and cytomegalovirus (CMV) was demonstrated in individuals with serious mental illness previously (142).

Increased levels of antibodies against food antigens has also been demonstrated in ASD (Table 1). In general, this disorder is characterized by high comorbidity of various gastrointestinal abnormalities e.g., constipation, diarrhea, reflux, esophagitis, gastritis, duodenitis, enterocolitis, lymphoid nodular hyperplasia, increased intestinal permeability, impaired detoxification (for example, defective sulfation of phenolic amines), SIBO, dysbiosis with bacterial overgrowth and yeast overgrowth (143–145). For instance, patients with ASD had increased parameters of intestinal permeability measured with lactulose/mannitol ratio (LA/MA) and they had elevated levels IgG to AGA, deamidated gliadin and IgG to casein (97). Moreover, increased concentrations of IgA antibodies to casein, lactalbumin and β-lactoglobulin, and IgG and IgM to casein were demonstrated in infantile autism and 8 weeks of an elimination diet identified by a positive skin test, resulted in marked improvement in behavioral symptoms (98). Furthermore, ASD patients had increased concentrations of zonulin, a physiological regulator of gut epithelium permeability via modulation of tight junctions opening between enterocytes (99). Also, Fiorentino et al. demonstrated in a *post mortem* study that 75% of ASD patients had reduced expression of components of intestinal tight junctions (claudin-1, occluding, and tricellulin), and 66% of patients had elevated expression of pore-forming claudins (claudin−2,−10,−15) compared to the control group (100). Moreover, in the same study, brain samples from patients with ASD revealed alterations in genes expression related to BBB stability, coupled with elevated neuroinflammation. However, patients with ASD may exhibit additional dysfunction of GI tract, which adds to the complexity of gut-brain-axis involvement in this disorder. For instance, decreased activity of carbohydrate digestive enzymes (disaccharidases or glucoamylase) was found in 58.3% of children with ASD (146) and multiple studies demonstrated association between disaccharidases deficiencies and intestinal inflammatory changes (147). The most frequent finding was a low lactase level. This enzyme has a role in the hydrolysis of lactose to glucose and galactose, and the latter is essential for the synthesis of brain galactolipids. Consequently, malabsorption of disaccharides is believed to play a role in the behavioral problems observed in non-verbal ASD patients. Also, decreased activity of GI enzymes e.g., dipeptidyl peptidase IV (DPPIV) has been suggested to be the cause of inadequate digestion of caseins including casomorphins and glutens including gliadomorphins in ASD (103, 148, 149) and those exorphins were demonstrated to exhibit pro-inflammatory properties (103, 150). “Leakiness” of both the intestinal and blood-brain barriers, observed in autistic patients, could result in easier access of neuroactive peptides and food derived antigens to the CNS which could have pro-inflammatory and neurobehavioral consequences. Also, decreased activity of DPP IV was demonstrated in depressed patients and DPP IV activity correlated with immune-inflammatory markers such as, number of CD4^+^T cells and CD4^+^/CD8^+^ T cell ratio (151, 152). Interestingly, therapeutic effects of an enzyme-based therapy for autism have also been reported and are believed to be due to the improvement of digestion of caseins, glutens and exorphins (153, 154).

## Molecular mimicry and antigenic covalent binding—a “trojan horse” of psychiatric autoimmunity?

A role of increased gut barrier permeability in the pathogenesis of autoimmune disorders has previously been described by Fasano et al. (155–157). Consequently, increased intestinal permeability was demonstrated in various autoimmune disorders e.g., celiac diseases, type 1 diabetes, asthma, multiple sclerosis, inflammatory bowel diseases, ankyloses spondylitis, and it is believed that gut-derived molecular mimicry could be a pathogenic factor of autoimmunity observed in those conditions (Figure 1). Various autoimmune disorders are known risk factors of major depression, schizophrenia, and psychotic disorders (158, 159) and comorbidity of autoimmune diseases is associated with a 45% increased risk of schizophrenia (160). Moreover, it is believed that autoantibodies could play a significant role in the pathogenesis of depression and that autoimmune and depressive disorders may share common pathogenic factors (161–166). Interestingly, intracerebroventricular injection of human anti-ribosomal P antibodies induced depressive behavior in mice (167). Also, in major depression and schizophrenia, increased concentrations of various autoantibodies to cellular proteins e.g., α7 nicotinic and dopamine receptors, cardiolipin, parietal cells (PCA), smooth muscle actin, antinuclear (ANA) and anti-thyroid gland (TGA) was demonstrated (168–170). Presence of serotonin autoantibodies was also revealed in patients with schizoaffective psychoses, chronic alcoholism and rheumatoid arthritis (171). In both schizophrenia and mood disorders, increased levels of autoantibodies to hypothalamus, hippocampus and cerebellum, and anti-nuclear antibodies was demonstrated (172). Also the presence of various other autoantibodies in schizophrenia has been previously reviewed (173). Recently, there has been a lot of scientific attention focused on neurologic and psychiatric manifestations related to various cell surface autoantibodies such as antibodies to N-methyl-D-aspartate glutamate receptor (NMDAR), α-amino-3-hydroxy-5-methyl-4-isoxazolepropionic acid receptor (AMPAR), voltage-gated potassium channel (VGKC), γ-aminobutyric acid-B receptor (GABA_B_R), the glycine receptor (GlyR) and metabotropic glutamate receptor 5 (mGluR5) (174). These antibodies could be associated with cancer, however more commonly they are non-paraneoplastic with the source of autoimmunity remaining unknown (175, 176). Co-occurrence of these autoantibodies was shown to be associated with various psychiatric symptoms e.g., psychosis, mania, agitation, emotional lability, anxiety, aggression, compulsive behavior, personality change, confusion, memory impairment, and amnesia (174). Antibodies to the NMDA receptor and to the VGKC were described in patients with schizophrenia (177–179). Respectively, Lennox et al. suggested that a sub-group of patients with a diagnosis of schizophrenia may actually suffer from undiagnosed NMDAR encephalitis (180). Interestingly, it was demonstrated that GI inflammation and increased intestinal permeability caused by *T. gondii* infection, a known risk factor of schizophrenia, resulted in the development of anti-NMDA receptor antibodies in laboratory animals. This infection also resulted in increased levels of anti-gluten and anti-casein IgG antibodies along with increased concentrations of complement factors which play a crucial role in neurodevelopment and neuronal pruning (42). In a recent breakthrough research, which clearly links gut-derived antigens with neuronal autoimmunity, Lambert and Vojdani demonstrated that patients with antibody reactivity to specific food proteins had higher co-occurrence of various tissue antibodies compared to controls without such food reactivities (181). More precisely, 35% of the control group (negative for IgG against glutens) and 64% of patients (positive for IgG against gluten) were reactive against tissues. Thirty percent of the control group (negative for dairy proteins antibodies) and 73% of patients (positive for dairy antibodies) were reactive against tissues. Twenty two percent of the control group (negative for IgG against wheat germ agglutinin—WGA) and 76% of patients (positive for IgG against WGA) were reactive against tissues. Furthermore, authors demonstrated that of all three groups of food antigens assessed (gluten, dairy, and lectin/agglutinin family proteins), autoimmune reactivity to neurological tissues was the highest in the patient group. It should be noted that this study did not provide specific information on patients' inclusion criteria and their diagnoses, therefore similar research in psychiatric patients is required.

Analogous immune mechanisms were also described in ASD where concomitant increase of IgG, IgM, IgA to gliadin, and cerebellum was demonstrated in more than 80% of autistic patients (101) (Table 1). Furthermore, the same study revealed a cross-reactivity between gliadin and cerebellar peptides. Moreover, it was suggested that autoimmunity due to bacterial infections and exposure to milk antigens may be a pathogenic factor in autism.

Voidani et al. also demonstrated that in individuals with predisposing HLA molecules, infectious agents including superantigens [for e.g., bacterial streptokinase (SK)], heat shock protein (HSP-60), dietary proteins (for e.g., gliadin and casein), and xenobiotics [for e.g., ethyl mercury (thimerosal derivate)] bind to different enzymes or cell surface receptors e.g., CD26 (DPP-IV) and CD69, and induce autoantibodies against HLA peptides (103, 148) (Table 1). In this study a significant percentage of ASD children developed anti-SK, anti-gliadin and casein, anti-ethyl mercury antibodies concomitantly with anti-CD26, anti-CD69 autoantibodies. Furthermore, adding SK, gliadin, casein, ethyl mercury to CD26 or CD69 resulted in 28–86% inhibition of CD26 or CD69 binding to anti-CD26 and anti-CD69 antibodies.

So far, two main mechanisms have been identified for food protein-induced autoimmunity in various tissues; firstly molecular mimicry, also known as cross-reactivity and secondly, covalent binding of food derived lectins and agglutinins to human tissues (181) (Figure 1). In molecular mimicry, there is case of “mistaken identity” between specific food antigens and human tissue due to high molecular homology e.g., amino acid homology of gliadin or dairy proteins with human tissue. In some scenarios (e.g., increased intestinal permeability) (Figure 1), antibodies against these food antigens are produced and the immune system can “mistake” these mimicking antigens for host tissue and react against it. For example, such reactions between gliadin and milk proteins with cerebellar tissue, and myelin have been reported previously (101, 182–184). In case of the second mechanism mentioned above, numerous plant lectins and agglutinins can covalently bind to various tissues and in response, the immune system may react against the new structure, as well as the surrounding tissue (181).

## Intestinal microbiota—new instrument in psychiatric treatment and diagnosis

Another component of the microbiota-gut brain axis with a crucial role determining intestinal permeability and immunity of both the GI tract and the CNS is intestinal microbiota. In the last few years, there has been significant progress in our understanding of the role of bacteria in brain function and behavior and these mechanisms have been reviewed extensively elsewhere (185–189). Microbiota play a significant role in maintaining the psycho-neuro-immunological balance by various mode-of-actions, such as the modulation of the immune and neuroendocrine systems, e.g., hypothalamic-pituitary adrenal axis (HPA), changes of the tryptophan (TRP) metabolism in the serotonin and kynurenic axes, production and metabolism of multiple neuroactive compounds e.g., short-chain fatty acids (SCFAs) and neurotransmitters. Beneficial bacteria also influence neurogenesis and the expression of neurotransmitters' receptors in the CNS (186, 187). Microbiota are also believed to be key regulators of neuroinflammation and to modulate mucosal innate and adaptive immune responses during infection, inflammation and autoimmunity (188). For instance, it was demonstrated that gastrointestinal microbiota have a significant function in the maturation and immune function of microglia (190). These bacteria also influence blood-brain barrier permeability (191). Furthermore, as natural guardians of the gut epithelium, intestinal microbiota, have a crucial role in the maintenance and modulation of gut epithelium barrier and in the regulation of various gut-associated lymphoid tissue (GALT) functions (29, 192). Key downstream effects of these beneficial microbes include their ability to decrease concentration of pro-inflammatory cytokines and the nuclear factor, NF-κB, increase concentrations of anti-inflammatory cytokines, and changes in tryptophan, and kynurenines levels (192–196). Since pro-inflammatory cytokines, NF-κB, and zonulin have a crucial role in increase of intestinal permeability, various microbiota, due to ability to modulate those parameters, have a protective effects on intestinal barrier (192–194, 197). Those bacteria have a beneficial influence on the composition of intestinal tight junctions proteins, inhibit adherence of pathogens to intestinal barrier, increase mucin production by epithelial goblet cells, increase secretory IgA (sIgA) and antimicrobial β-defensin secretion into the luminal mucous, what enhances intestinal barrier (198).

There is growing evidence to support the therapeutic effects of microbiota and probiotics on the symptoms of anxiety, low mood and depression, CFS, and cognitive functions (186, 199–215) and beneficial bacteria have recently earned a general name of psychobiotics (216).

Altered composition of gut microbes was demonstrated in various psychiatric disorders including CFS (79), MDD (217–220), ASD (221–225), schizophrenia and bipolar disorder (226), and alcoholism (38). In MDD altered proportions of Prevotella and Klebsiella bacterial genus were consistent with the Hamilton depression rating scale (220). Additionally, fecal microbiota transplant from patients with major depression to germ free (GF) mice resulted in depression-like behavior in recipient mice (227). Small intestine bacterial overgrowth (SIBO) is another abnormality of intestinal flora observed in ASD and alcoholism (145, 228). It was hypothesized that these alterations could play a significant role in psychopathology and assessment of flora composition could become a clinical marker in psychiatry. Interestingly, psychiatric pharmacotherapy was shown to influence composition of intestinal microbiota. For example, antipsychotic medication such as olanzapine and risperidone have been shown to modify gut flora and it was further demonstrated that weight gain, often observed in patients during such treatment, was secondary to altered of gut microbiota by the antipsychotics (229–231). Moreover, olanzapine-induced metabolic dysfunction in rats was attenuated by antibiotic administration (232). Consequently, probiotics administration could provide a novel therapeutic strategy for the prevention or reversal of weight gain following antipsychotic treatment.

## Future perspectives—the paradigm shift in psychiatry emerging

We are witnessing a truly interesting time for psychiatry and neuroscience. The last two decades of research in the field of psycho-neuro-immunology have provided us with an increased understanding of the role of immunity in psychiatric disorders. Now, with the involvement of the microbiota-gut-brain axis, the second stage of this inevitable paradigm shift in psychiatry has begun. In the old paradigm, psychiatry was “starting” when “all” physical abnormalities (besides clearly organic disorders) were excluded. Currently, the split between purely psychological vs. medical background of psychiatric disorders is dissolving in response to improved understanding of psycho-neuro-immune interactions between psyche and soma. Having recognized that vast amounts of psychiatric symptomatology may have an immune, autoimmune and/or gut-derived background, one wonders when we face the separate nosological entities and when we face various manifestations of underlying immune processes. Professor Ronald S. Smith, the precursor of the inflammatory hypothesis of depression, stated the following in his sadly unfinished book, “Cytokines & Depression. How your immune system *causes* depression.” While describing immune pathogenesis of depression, he referred to the First Edition of Encyclopaedia Britannica and challenged the current approach to major depression diagnosis. In this edition, published in 1771, 37 different subtypes of “fever disease” were described as separate disorders. Smith wrote, “*Eventually it was understood that fever in not one disease nor 37 kinds of fever diseases, but rather it is a trustworthy* ***universal sign of acute immune system activation****. Fever is a sign of* ***acute****immune system activation, regardless of any other signs, symptoms or diseases that it may be associated with. After this realization, fever was no longer a bewildering and complex disease, but instead, a simple, direct and easily understood signal of acute immune activation”* (233). On the other hand, Susannah Cahalan in her New York Times Bestselling autobiography, “Brain on Fire: My Month of Madness,” describes her own horrifying experience of anti-NMDA receptor encephalitis. Initially, due to various psychotic symptoms, a diagnosis of bipolar affective disorder, schizophrenia, or schizoaffective disorder was suggested, however after further investigation, she was diagnosed with aforementioned encephalitis. After all, Cahalan was diagnosed with and treated for a neurologic, autoimmune disorder, and a psychiatric diagnosis was rejected. However, this still provokes the question; how many psychiatric patients suffer from similar autoimmune conditions with lesser manifestations of neurologic symptoms? When are we dealing with a psychiatric manifestation of “organic” disorder and when is it a “purely” psychiatric one? Maybe, as Smith suggested, we are more commonly witnessing psychiatric manifestations of immune system activation. The GI tract is the largest immune organ in the human body and also the biggest surface area of interaction between the internal and external environment. Taking this into consideration and in light of the discussion included in this review; the GI tract will undoubtedly have a major impact on psychiatric symptoms and treatment.

Since changes in exposure to food antigens have been shown to modulate immune response, further research using elimination diets in a subgroup of patients expressing increased levels of food-specific antibodies would be of value. The benefits of dietary interventions in psychiatric patients have been described previously (234). Moreover, the therapeutic effects of elimination diet were described in neurologic and GI disorders such as irritable bowel syndrome, Crohn's disease and migraine, and improvement in symptoms was believed to be related to decreased levels to food-specific IgG as a result of decreased exposure to food antigens (235–240). Also, augmenting psychiatric pharmacotherapy with digestive enzymes and interventions known to positively influence the gut barrier may have a positive effect on the microbiota-gut-brain axis and psychiatric symptomatology. For instance, supplementation of curcumin and zinc, which are both excellent “tightners” of gut barrier, along with pre and probiotics might improve symptoms of psychiatric and co-morbid inflammatory and autoimmune disorders (241, 242). Both turmeric and zinc exhibit anti-inflammatory effects and their anti-depressant effects were previously demonstrated (243, 244). Curcumin has a positive effect on gut barrier due to its ability to decrease levels of TNFα, a pro-inflammatory cytokine which negatively influences tight junctions and increases intestinal and BBB permeability (35, 36, 60, 64–66, 245). This cytokine is involved also in activation of kynurenic enzyme indoleamine 2,3 dioxygenase (IDO) which diverts tryptophan from serotonin pathway toward detrimental kynurenines. Consequently, curcumin decreases IDO activity (246). Moreover this herb is a well-known inhibitor of NF-κβ signaling involved in inflammatory response and increased permeability of both intestinal and blood-brain barriers (247).

To conclude, the assessment of intestinal permeability, gut derived immunity, immunoglobulins against various food, microbial, viral and parasitic antigens, and assessment of intestinal flora composition may be valuable in psychiatric diagnosis and therapy. Also, modification of intestinal flora may prevent metabolic side effects related with antipsychotic treatment. Finally, supplementation of probiotics or other interventions to positively influence the intestinal barrier could be used as a preventive measure of exposure to stress and its detrimental consequences.

## Author contributions

LR and AS designed and wrote the first version of manuscript and performed literature search. LR wrote the final version of manuscript, prepared figures and tables.

### Conflict of interest statement

The authors declare that the research was conducted in the absence of any commercial or financial relationships that could be construed as a potential conflict of interest.
